# The Application of Artificial Intelligence in Brucellosis: Diagnosis, Genomic Analysis, Epidemiological Prediction and Drug Discovery

**DOI:** 10.3390/ani16203241

**Published:** 2026-10-15

**Authors:** Xiaofang Liu, Haifeng Wen, Tengyu Wang, Hao Liu, Mingming Xie, Yajie Luo, Luchao Zuo, Chunxia Liu, Dong Zhou, Wenlong Wang

**Affiliations:** 1 College of Veterinary Medicine, Inner Mongolia Agricultural University, Hohhot 010011, China; liushine1113@163.com (X.L.); 15024959754@163.com (H.W.); w1281410904@163.com (T.W.); 18586281243@163.com (H.L.); 19804899246@163.com (M.X.); luoyajie595@163.com (Y.L.); 13393107344@163.com (L.Z.); 2 Key Laboratory of Animal Disease Clinical Diagnosis and Treatment Technology, Ministry of Agriculture and Rural Affairs, College of Veterinary Medicine, Inner Mongolia Agricultural University, Hohhot 010011, China; 3 College of Life Sciences, Inner Mongolia Agricultural University, Hohhot 010011, China; lcx.imau@imau.edu.cn; 4 College of Veterinary Medicine, Northwest A&F University, Yangling 712100, China

**Keywords:** artificial intelligence, *Brucella*, brucellosis, prevention and control

## Abstract

Brucellosis, a globally significant zoonosis caused by *Brucella*, poses a substantial impediment to the sustainable development of the livestock industry and threatens public health security. Traditional control and prevention strategies for brucellosis suffer from fundamental limitations. These include the time-consuming, biosafety risks associated with bacterial culture and the difficulty in differentiating between natural infection and vaccine-induced immunity with serological tests. There remains a challenge in accurately distinguishing vaccine strains from wild-type strains via genotyping methods. Furthermore, current practice relies on manual reporting and empirical judgment without real-time data support. Consequently, these challenges could hinder early detection and reduce the efficiency of large-scale screening, which leads to imprecise outbreak tracing and fails to meet the practical demands of modern livestock production for rapid diagnosis, intelligent prevention and control. In recent years, artificial intelligence (AI) and machine learning (ML) technologies have been widely applied in the field of veterinary microbiology, which could offer novel technical pathways for the prevention and control of brucellosis. This paper reviews the latest research progress in the application of AI for the rapid diagnosis of brucellosis, genomic analysis and antimicrobial resistance prediction of *Brucella*, epidemiological risk early warning and the development of drugs and vaccines. We further analyze the current limitations of AI in brucellosis research including the scarcity of high-quality, annotated datasets, insufficient model interpretability and low efficiency in on-site translation and implementation. The paper provides an outlook on the future development of AI-based intelligent prevention and control systems for brucellosis, which offer a theoretical reference and innovative perspectives for the fundamental research and clinical application of AI technologies. To the best of our knowledge, this is the first review to integrate AI applications across the entire chain of brucellosis prevention and control and to propose a conceptual framework for AI-driven intelligent brucellosis management.

## Simple Summary

Brucellosis is a globally important zoonotic disease caused by *Brucella* that seriously hinders the sustainable development of the livestock industry and threatens public health security. Traditional prevention and control strategies face fundamental limitations. Bacterial culture is time-consuming and carries biosafety risks; serological tests struggle to distinguish natural infection from vaccine-induced immunity; and genotyping methods have difficulty accurately separating vaccine strains from wild-type strains. Current practice also depends on manual reporting and empirical judgment without real-time data support. As a result, these challenges hinder early detection and reduce the efficiency of large-scale screening, which leads to imprecise outbreak tracing and delayed responses and fails to meet the practical demands of modern livestock production for rapid diagnosis and intelligent prevention and control. In recent years, artificial intelligence (AI) technologies have been widely applied in veterinary microbiology, which offer novel technical pathways for brucellosis prevention and control. This paper reviews the latest research progress in using AI for rapid diagnosis of brucellosis, genomic analysis and antimicrobial resistance prediction of *Brucella*, epidemiological risk early warning and the development of drugs and vaccines. It further analyzes the current limitations of AI in brucellosis research including the scarcity of high-quality annotated datasets, insufficient model interpretability and low efficiency in on-site translation and implementation. Finally, the paper provides an outlook on the future development of AI-based intelligent prevention and control systems for brucellosis, which offers a theoretical reference and innovative perspectives for fundamental research and clinical application of AI technologies.

## 1. Introduction

Brucellosis, a zoonotic infectious disease caused by bacteria of the genus *Brucella*, poses a significant global health burden with an estimated 160,000 to 210,000 human cases reported annually worldwide [1]. *Brucella* spp. are facultative intracellular, Gram-negative strain that cause brucellosis. This disease is one of the most widespread zoonotic diseases worldwide and a disease of major socioeconomic importance. The three *Brucella* species of greatest zoonotic concern are *B. melitensis*, *B. abortus*, and *B. suis* [2,3,4]. Currently, the disease predominantly infects domestic livestock such as pigs, cattle and sheep. In affected animals, brucellosis frequently manifests as reproductive disorders including abortion, stillbirths and infertility. In humans, *Brucella* infection is usually acquired via direct contact with infected animals or ingestion of contaminated animal-derived foods, leading to a chronic, debilitating illness characterized clinically by relapsing fever, arthralgia, and multi-system involvement [5]. The regional endemicity of brucellosis not only incurs economic losses to the livestock industry, but also severely influences public health security and social stability. Consequently, it is classified as a key controlled disease in the field of veterinary public health [6]. The current conventional technical framework for brucellosis control and prevention relies on a suite of diagnostic and investigative methods. These include pathogen isolation and microscopic examination, serological testing, molecular biological identification, manual genomic analysis and empirical epidemiological investigations [7,8]. However, each of these conventional approaches is subject to inherent limitations. Bacterial isolation requires stringent biosafety conditions and poses a biohazard risk to laboratory personnel [9]. The interpretation of conventional microscopy and serological assays depends on technician judgment and assay-specific factors. And reported misdiagnosis rates vary widely, depending on operator experience, endemicity, and diagnostic thresholds. Serological cross-reactivity and low sensitivity in early or low-bacterial-load samples are well-recognized limitations. Similarly, while traditional manual genomic analysis based on comparative annotation is time-consuming and difficult to scale for high-throughput strain typing, this reflects constraints of throughput, standardization, and accessibility rather than an inherent inability to differentiate strain types [7]. Moreover, conventional epidemiological early-warning models often fail to integrate multidimensional data such as environmental factors, livestock management practices, and outbreak information. This limitation delays risk predictions and impedes the implementation of proactive, precise prevention and control strategies [10]. Anti-*Brucella* drug and vaccine development remains largely dependent on traditional screening approaches whose time-intensive and costly nature impedes the rapid iteration and innovation of brucellosis control tools [11].

A diverse array of AI algorithms has been progressively integrated into the precision prevention and control of bacterial infections with the rapid advancement of big data and deep learning (DL) technologies [12]. Intelligent models, exemplified by ML, convolutional neural networks (CNNs) and long short-term memory (LSTM) networks, have demonstrated considerable capability in efficiently mining multifaceted data types pertinent to brucellosis, including image-based detection data, genomic feature information, epidemiological time-series data, and drug molecular profiles. These approaches have shown promising application prospects across a spectrum of domains, encompassing intelligent diagnosis, strain typing and antimicrobial resistance prediction, epidemic risk early warning, pathogenesis elucidation, and the development of novel biological agents [13,14,15]. Collectively, these AI-driven strategies offer intelligent solutions to overcome the technological bottlenecks that have long constrained conventional brucellosis prevention and control measures.

**Figure 1:**
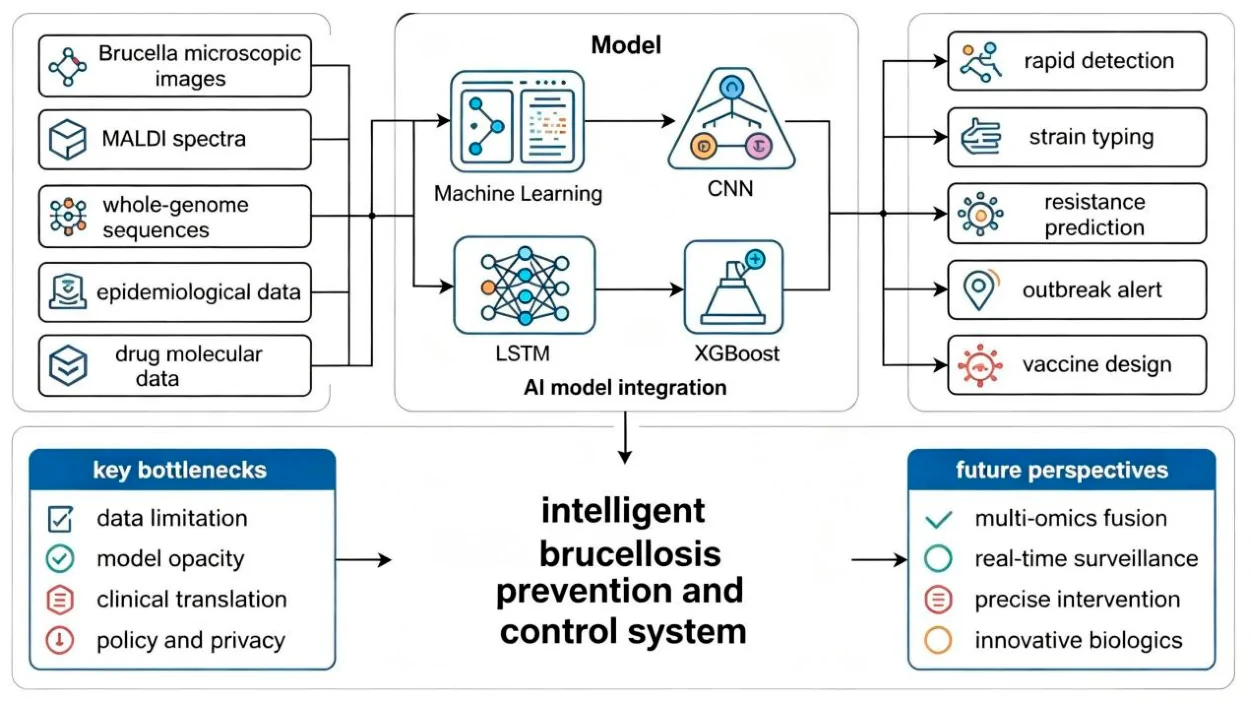
AI models may increase the likelihood of detecting brucellosis cases.

This review synthesizes current research progress on the application of AI in brucellosis, covering intelligent diagnostics, genomic analysis, antimicrobial resistance prediction, epidemiological risk assessment, pathogenesis mining and biopharmaceutical development. Furthermore, this article critically analyzes the core bottlenecks that impede the in-depth and broader application of AI in brucellosis research, proposing future developmental trajectories for intelligent prevention and control technologies. This work aims to provide novel insights for the innovation and advancement of a precision-oriented brucellosis prevention and control system. This is the first review to integrate AI applications across brucellosis prevention and control, spanning intelligent diagnostics, genomic analysis, antimicrobial resistance prediction, epidemiological risk assessment, pathogenesis mining and biopharmaceutical development. This article critically analyzes the core bottlenecks that impede the broader translation of AI in brucellosis research, including the scarcity of high-quality annotated datasets, insufficient model interpretability, low efficiency in on-site implementation, and proposes future developmental trajectories for intelligent prevention and control technologies. This work aims to provide an integrated, precision-oriented AI framework and novel insights for brucellosis prevention and control systems of brucellosis (Figure 1) and Table 1.

This figure illustrates an artificial intelligence system framework for intelligent brucellosis prevention and control. The system integrates multiple data sources, including *Brucella* microscopic images, MALDI spectra, whole-genome sequences, epidemiological data and drug molecular data, and feeds them into an AI model that combines algorithms such as machine learning, CNN, LSTM, and XGBoost. Through this AI model integration, the system can achieve functions such as rapid detection, strain typing, resistance prediction, outbreak alerts and vaccine design. Although it currently faces key bottlenecks such as data limitation, model opacity, difficulties in clinical translation, and policy and privacy concerns, the system is expected to further enhance brucellosis prevention and control capabilities in the future through approaches such as multi-omics fusion, real-time surveillance, precise intervention and innovative biologics.

## 2. Materials and Methods

Databases searched: We searched databases including PubMed, Web of Science and Scopus. Search terms and combinations: The search terms used included “brucellosis,” “*Brucella*,” “artificial intelligence,” “machine learning,” “deep learning” and “AI”. Inclusion and exclusion criteria: Inclusion criteria were studies involving the application of AI in brucellosis. Process used to identify eligible studies: Two authors independently screened the literature, first by title and abstract, and then by full text. Disagreements were resolved through discussion or arbitration by a third author.

## 3. Application of AI in Rapid Diagnosis of Brucellosis

**Figure 2:**
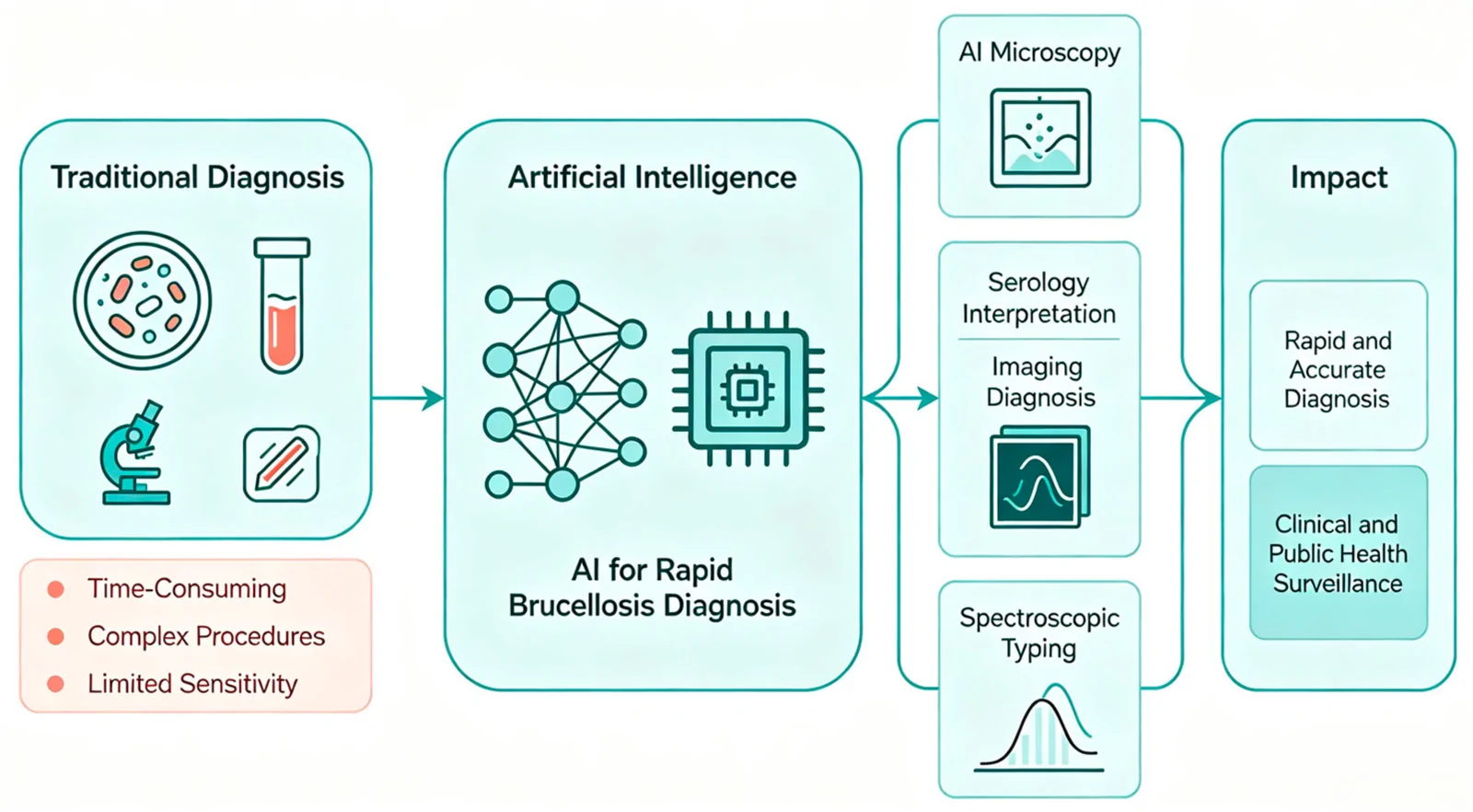
Application of AI in rapid diagnosis of brucellosis.

The application of artificial intelligence (AI) in the rapid diagnosis of brucellosis has demonstrated considerable potential. brucellosis, as one of the most common zoonotic diseases worldwide, is conventionally diagnosed using bacterial culture and serological tests. However, these traditional approaches are constrained by prolonged turnaround times, complex operational procedures and suboptimal sensitivity. The introduction of AI technologies could offer new avenues for diagnosis of brucellosis. The primary manifestations include AI-assisted microscopic image recognition, intelligent interpretation of serological assay results, AI-driven imaging diagnosis and the integration of spectroscopic techniques with AI for *Brucella* typing and identification as well as for the radiological assessment of pathological changes. Collectively, these AI-based modalities could provide reliable technical support for the rapid and accurate diagnosis of *Brucella* infections and are expected to play a significant role in both clinical management and public health surveillance (Figure 2).

This figure illustrates how artificial intelligence (AI) is revolutionizing the diagnostic workflow for brucellosis and enhancing its public health impact. Traditional diagnostic methods for brucellosis suffer from limitations including being time-consuming, involving complex procedures and having limited sensitivity. To address these issues, the system introduces artificial intelligence specifically for rapid brucellosis diagnosis. AI technology is applied to several key areas, including AI microscopy, serology interpretation, imaging diagnosis and spectroscopic typing. Through these AI-driven diagnostic tools, the system ultimately enables rapid and accurate diagnosis, exerting a profound positive impact on clinical practice and public health surveillance.

### 3.1. AI-Assisted Microscopic Image Recognition Technology

Microscopic examination of stained bacterial smears remains the most fundamental and intuitive preliminary screening method for *Brucella*. Conventional manual microscopy requires technicians to visually identify bacterial morphology, perform cell counting and determine positive or negative results. This process is subject to subjective bias, is time-consuming, demands stringent laboratory conditions, and is poorly suited to high-throughput sample testing. In recent years, DL-based image recognition techniques have been progressively adopted for the intelligent discrimination of pathogenic bacteria in microscopic images. The accuracy of bacterial species identification can be improved through a label-free surface-enhanced Raman scattering (SERS) analytical pipeline integrated with ML [16]. Object detection algorithms such as convolutional neural networks (CNNs) and the YOLO (You Only Look Once) series have been proven effective for automatic feature extraction and detection of cells and pathogens in medical microscopic images. Large-scale annotated microscopic image datasets have enabled such models to achieve automatic localization, quantitative counting and binary classification of target microorganisms, while effectively eliminating interfering elements including contaminating bacteria, background debris and staining artifacts in *Mycobacterium tuberculosis* [17]. The YOLOv4 network was applied for automatic detection and growth-phase classification in microscopic images in *Escherichia coli* [18]. Similarly, YOLOv5, YOLOv7, and YOLOv8 have been employed for object detection on Gram-stained smear images of 14 bacterial species [19]. This technical paradigm provides a feasible solution for intelligent microscopic analysis of *Brucella* and other pathogens. These studies have demonstrated that AI-based microscopic examination models could exhibit higher accuracy and sensitivity compared to manual interpretation. This approach could be particularly well-suited for epidemiological surveillance in large-scale livestock operations and large-volume screening at primary veterinary diagnostic facilities.

### 3.2. AI-Assisted Intelligent Interpretation of Serological Assays

The Rose Bengal Plate Test (RBPT) and colloidal gold immunochromatographic strips are both the most commonly employed techniques for rapid on-site screening of brucellosis. However, the interpretation of serological results relies on visual observation of color reactions and the degree of agglutination or precipitation, which are susceptible to operator-dependent errors and lead to false-positive and false-negative outcomes [20].

Recent research has explored the integration of ML with advanced sensing platforms to achieve objective and intelligent serological interpretation. A photonic waveguide scattering microscopy technique was developed for the detection of anti-*B. abortus* antibodies in bovine serum by capturing images of scattering spots generated by agglutination reactions and training an artificial neural network (ANN) [21,22].

AI-based image processing techniques employ mobile terminals or industrial cameras to capture gray-scale values, color differences, and reaction-zone information from serological results, enabling quantitative discrimination models to be constructed for fully automated intelligent interpretation of the RBPT and colloidal gold strip tests. In contrast to manual qualitative judgment, AI-driven quantitative analysis can identify weakly positive samples that are difficult to distinguish by the naked eye, thereby improving the detection accuracy for specimens with low pathogen loads. Concurrently, this approach standardizes serological interpretation criteria and eliminates inter-operator variability. Furthermore, the integration of ML with lateral flow assays (LFAs) has been extensively investigated. Models such as convolutional neural networks (CNNs) can automatically extract signal features from smartphone-captured test strip images, enabling objective and quantitative readouts, thus effectively reducing human reading errors [23,24].

### 3.3. Integration of Spectroscopic Techniques and AI for Differential Typing of Brucella: Distinguishing Wild-Type Strains from Vaccine Strains

Discriminating between wild-type *Brucella* strains and vaccine strains has long been a critical challenge in the accurate laboratory diagnosis of brucellosis. Conventional serological assays exhibit limited capacity to differentiate naturally acquired infections from vaccine-induced immune responses due to cross-reactive antigens. Although single nucleotide polymorphism (SNP)-based digital droplet PCR enables reliable identification of wild-type *B.suin* S2 vaccine strains, their widespread application in field settings is constrained by high operational complexity, substantial cost and dependence on specialized instrumentation [25]. In response to these limitations, the synergy between spectroscopic fingerprinting and ML has emerged as a strategy for rapid, on-site *Brucella* subtyping. Raman spectroscopy can resolve distinctive molecular signatures derived from cell wall components, proteins and nucleic acids across different *Brucella* strains. Furthermore, the integration of surface-enhanced Raman scattering (SERS) with lateral flow immunoassay (LFIA) enabled simultaneous detection of anti-*Brucella* IgG and IgM antibodies within 15 min with quantitative readouts feasible via portable Raman instruments. Fourier-transform infrared (FTIR) spectroscopy has similarly exhibited remarkable discriminatory power. A recent large-scale investigation, encompassing 2925 FTIR spectra from 163 *Brucella* isolates representing diverse species and biovars, employed support vector machines (SVM) and artificial neural networks (ANN) to construct classification models. This model demonstrated sensitivity and specificity at the species l and biovar level, which inherently includes discrimination between vaccine and field strains resulting in excellent overall classification accuracy [26]. These findings substantiate that FTIR spectroscopy coupled with AI enables high-throughput, automated identification of *Brucella* at the species, biovar, and vaccine-versus-wild-type strain levels. In a separate study, FTIR spectroscopy combined with SVM was applied to diagnose bovine brucellosis and attained an accuracy in differentiating 40 infected sera from 40 healthy controls, with a total assay time of approximately 5 min [15]. Collectively, AI- and ML-based technologies offer advantages including rapid turnaround, non-destructive sample handling and low operational costs. These attributes effectively address the shortcomings of traditional typing approaches—namely, laborious procedures, protracted time-to-result and insufficient resolution between vaccinated and naturally infected individuals.

### 3.4. AI-Assisted Pathological Imaging Diagnosis

*Brucella* infection can induce characteristic pathological changes in animals including visceral granulomas, joint damage and reproductive system inflammation. Traditional imaging diagnosis relies on the subjective interpretation of radiologists based on clinical experience. However, brucellar spondylitis (BS) and tuberculous spondylitis (TS) exhibit overlapping imaging features, which make differential diagnosis challenging and prone to misdiagnosis or oversight. AI-based imaging recognition technologies offer a promising solution to this dilemma. DL-based imaging diagnostic models have demonstrated significant advantages in the MRI-based differential diagnosis of brucellar spondylitis. BSMRINet, a fully automated diagnostic framework built upon ResNet and DenseNet architectures, is designed to detect and localize vertebral lesions on T2-weighted MRI images while simultaneously assessing paravertebral abscesses, soft tissue swelling and joint involvement. Its diagnostic performance surpassed that of senior radiologists with 10~15 years of experience [25]. In addition, a convolutional block attention module (CBAM) was integrated into the ResNeXt-50 architecture to construct a model based on sagittal T2-weighted images, which increased accuracy in distinguishing BS from TS compared to generic models such as ResNet50, GoogleNet and EfficientNetV2 [25]. Super-resolution-enhanced CT imaging was combined with DL radiomics employing ResNet34 for feature extraction to develop a comprehensive model [27]. Furthermore, a GoogleNet-based DL framework incorporating multimodal CT and MRI imaging has also been validated for effective discrimination between BS and TS. Collectively, these findings indicate that AI-based imaging recognition can automatically segment pathological foci in animal B-ultrasound, X-ray, CT and MRI images, quantify lesion extent and severity, and enable early screening of subclinically infected animals, thereby providing a reference for early clinical intervention and further refining the comprehensive diagnostic system for brucellosis. In recent years, multimodal intelligent detection strategies have achieved progress in the rapid diagnosis of brucellosis. A biosensing technology combining recombinase polymerase amplification (RPA) with CRISPR/Cas12 is developed, which achieves a fluorescence-based detection limit as low as 1 copy/μL compared with conventional quantitative PCR [28]. Another study establishes a one-pot isothermal detection assay based on RPA-CRISPR/Cas12b targeting the species-specific BCSP31 conserved sequence in *Brucella*. The fluorescence and lateral flow detection limits were 3.65 × 10^1^ and 3.65 × 10^2^ copies/reaction, but conventional PCR results were achieved across 61 clinical samples, with the entire procedure completed within 40 min [29]. Additionally, a rapid detection method also based on RPA-CRISPR/Cas12a attain a detection limit of 1 copy/μL and a turnaround time of less than 30 min in *Brucella* [30]. These RPA-CRISPR biosensing platforms offer the advantages of isothermal amplification. Coupled with lightweight AI quantitative models, this technology could improve sensitive on-site detection of *Brucella,* which effectively identifies low-burden latent infections and significantly improves the detection accuracy of subclinical brucellosis.

## 4. Application of ML in *Brucella* Genomic Analysis and Antimicrobial Resistance Prediction

**Figure 3:**
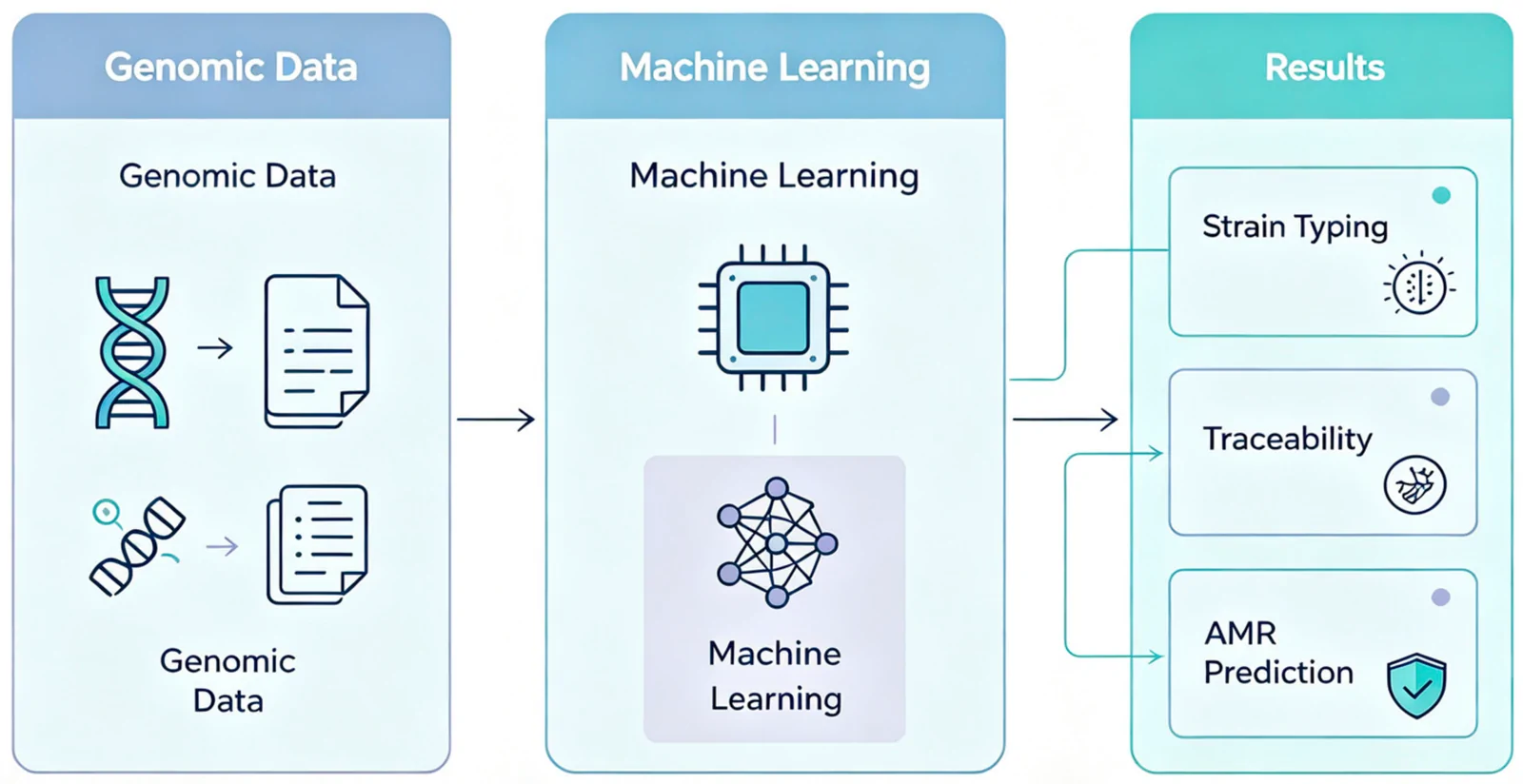
Application of machine learning in *Brucella* genomic analysis and antimicrobial resistance prediction. This figure illustrates the workflow of using machine learning to analyze genomic data and obtain specific results. First, in the “Genomic Data” stage, the system collects and processes genomic information such as DNA sequences and plasmids. Subsequently, this data is input into the “Machine Learning” module for in-depth analysis and model training. Finally, the machine learning model outputs three key “Results”: Strain Typing, Traceability, and AMR Prediction. Overall, this workflow demonstrates the complete transformation process from raw genomic data to machine learning analysis, and ultimately to specific clinical application results.

The widespread adoption of high-throughput sequencing technologies has generated massive genomic data for *Brucella*. Traditional genomic analysis relies on manual alignment, annotation, and statistical approaches, which struggle to fully exploit the underlying biological information embedded within big data. ML offers a powerful means to efficiently mine key genomic features including single nucleotide polymorphisms (SNPs), pangenomic content and core gene sequences. This tool demonstrated substantial value in *Brucella* strain typing, molecular traceability and antimicrobial resistance prediction (Figure 3). Interpretation of AI models specifically tailored for brucellosis should be developed with optimized algorithmic architectures and exploration of explainable ML approaches to clarify the biological significance of model feature variables. The integration of AI-based predictions with conventional experimental validation will enable the organic fusion of intelligent prediction and biological mechanisms.

### 4.1. Intelligent Strain Typing and Molecular Traceability of Brucella

Strain typing and epidemiological traceability are essential for understanding brucellosis transmission and guiding targeted interventions. Conventional molecular typing methods face some limitations. MLST can assign lineage designations but provides poor resolution due to declining whole-genome sequencing costs. PCR-sequencing- based typing using housekeeping genes has lost its economic advantage [31,32]. MLVA is operationally simple and useful for outbreak investigations and rapid strain verification, but its polymorphic loci vary considerably across geographic regions. Among Chinese *B. melitensis* strains, most VNTR loci in the MLVA-16 panel exhibit low or negligible polymorphism, undermining the method’s discriminatory power for local epidemic strains [33].

The introduction of ML has provided new avenues for traditional typing approaches. At the strain typing level, researchers have integrated ML algorithms with MALDI-TOF mass spectrometry. Through systematic analysis of mass spectra from 44 strains of *B. melitensis*, *B. abortus* and *B. suis*, a consensus feature selection strategy was employed to screen 16 differentially expressed peaks from a total of 500 mass spectral features. Supervised learning classifiers including random forest, support vector machine (SVM) and neural networks were subsequently constructed [34]. Similarly, efficient species-level typing of *Brucella* using MALDI-TOF mass spectrometry coupled with ML algorithms was accomplished [35].

With respect to strain traceability and transmission potential assessment, ML models have demonstrated enhanced analytical capabilities [36]. Although traditional core-genome maximum-likelihood phylogenies can delineate evolutionary relationships among different *Brucella* species, they are unable to effectively identify the transmission risk posed by emerging strains or to quantitatively evaluate the zoonotic potential of strains originating from different hosts [37]. To address this fundamental limitation, researchers innovatively combined pan-genome-wide association study (pan-GWAS) with ML. Based on whole-genome data from 991 *Brucella* strains, 268 feature genes associated with transmission potential were identified and an SVM-based predictive model was subsequently constructed [36]. This model provides qualitative evolutionary relationships and achieves a quantitative assessment of cross-host transmission potential in *Brucella*. This finding reveals that human-derived *B. melitensis* strains exhibit significantly higher transmission potential than bovine- or ovine-derived strains and that porcine-derived *B. suis* biovar 2 strains show elevated transmission risk compared to wild-boar-derived strains. The model’s generalizability shows high concordance with epidemiological reports in recent years. Further interpretation of key feature genes via the SHAP method identified that genes such as ABC transporter ATPase and urea transporter, which are closely associated with enhanced virulence and host adaptation, carried the highest weights in the model, thereby providing novel clues for unraveling the pathogenic mechanisms [36].

### 4.2. AI for Predicting Phenotypic Antimicrobial Resistance and Resistance Genes in Brucella

The increasing prevalence of antimicrobial resistance (AMR) in *Brucella* poses a significant challenge to the clinical management of brucellosis. Conventional antimicrobial susceptibility testing (AST) relies on prolonged bacterial culture and is time-consuming and labor-intensive. Many existing laboratory techniques fail to meet the urgent clinical demand for rapid therapeutic decision-making. The reliability of any genotype-to-phenotype prediction model depends fundamentally on the quality of the phenotypic labels used for training. For *Brucella*, AST presents particular challenges. CLSI breakpoints for *Brucella* species have not been formally established; most studies, including the reviewed work, extrapolate breakpoints from slowly growing bacteria such as *Haemophilus* spp. This extrapolation introduces uncertainty regarding whether observed MIC values truly correspond to clinical resistance. Furthermore, AST methodologies vary across studies—broth microdilution, E-test and agar dilution have all been employed with different incubation times and media compositions. ML offers a novel intelligent paradigm for high-throughput prediction of AMR in *Brucella*. A unified data standard system for intelligent *Brucella* detection, including strain typing and resistance prediction, is established to try to solve the current fragmentation and inconsistency of data through multi-center collaborative research frameworks. Leveraging large-scale genomic data coupled with matched phenotypic antimicrobial susceptibility testing (AST) profiles from *Brucella* isolates, this study employed ML algorithms including XGBoost, random forest, support vector machine (SVM) and logistic regression to screen for critical single nucleotide polymorphisms (SNPs) and functional genes associated with antimicrobial resistance. Subsequently, these identified genetic markers were utilized to construct predictive models for resistance phenotypes, which offer a scalable framework for genotype-based resistance surveillance [36]. A total of 636 clinical isolates of *B. melitensis* collected across China from 2010 to 2021 employed whole-genome sequencing, transcriptome sequencing and neural network-based prediction to analyze the resistance trends toward trimethoprim-sulfamethoxazole (SXT) [38]. This finding revealed SXT resistance was increased among Chinese *Brucella* isolates with distinct SNP signatures identified. The knockout of the glycoside hydrolase family 25 (GH25) gene was associated with SXT resistance and significantly altered energy metabolism in *Brucella,* which uncovered the metabolic adaptation driving resistance [38]. The model enables rapid prediction of phenotypic resistance to commonly used antibiotics and multi-drug resistance risks in *Brucella*. Furthermore, a strategy integrating pan-genome-wide association study (pan-GWAS) with ML has been applied to identify signature genes linked to *Brucella* transmission potential, resulting in the identification of 268 relevant genes. An SVM-based prediction model was subsequently developed to enable quantitative assessment of cross-host transmission risks. This analytical framework is readily transferable to the discovery and functional characterization of AMR-associated genes. The emerging genomic language model resLens achieved a groundbreaking innovation in bacterial AMR research [39]. Departing from conventional database-dependent resistance gene detection approaches—such as ResFinder and CARD—resLens fine-tunes a pre-trained DNA language model to recognize resistance genes by harnessing its intrinsic genomic representational capacity. The study demonstrated that this method achieved classification performance comparable or superior to alignment-based tools across multiple evaluation scenarios. Notably, under conditions where resistance gene sequences exhibited low similarity to known sequences in reference databases, resLens outperformed traditional alignment tools, successfully identifying gene families such as blaADC and ANT that are challenging to detect using conventional tools. In genotype–phenotype prediction, the resLens genomic language model, while demonstrating impressive performance on held-out gene families, was evaluated primarily on sequences with no *Brucella*-specific validation. For the brucellosis-specific models, the absence of clearly reported independent validation means that reported performance metrics—even if they appear robust in cross-validation—may not reflect true predictive accuracy on new isolates. This model can uncover resistance genes that evade routine annotation pipelines, effectively address critical gaps in existing resistance gene annotation frameworks and open new avenues for deciphering previously unknown regulatory mechanisms underlying AMR in *Brucella*.

## 5. AI-Based Epidemiological Prediction and Risk Assessment for Brucellosis

The epidemic transmission of brucellosis is governed by a complex interplay of multiple factors including climatic and environmental conditions, livestock stocking levels, herd immunity status and the movement of animal products. Traditional epidemic early-warning models such as SARIMA are capable of handling non-stationary time-series data and capturing both trend and seasonal components. However, this model constrains their ability due to their inherent reliance on linear assumptions and cannot fully integrate multidimensional nonlinear influencing factors, resulting in suboptimal predictive accuracy and timeliness.

DL algorithms, particularly long short-term memory (LSTM) networks due to their specialized gating mechanisms, have demonstrated marked advantages in infectious disease and effectively solve problems of the vanishing and exploding gradient in time-series data. Previous studies have demonstrated that ARIMA models are successful in predicting brucellosis incidence trends. Furthermore, the convolutional LSTM (ConvLSTM) can integrate spatiotemporal information and has achieved superior predictive performance compared with standard LSTM. The applicability of ConvLSTM, random forest (RF) and LSTM models is evaluated for brucellosis forecasting in Shaanxi Province, China. This result shows that ConvLSTM, as the optimal model, achieves a root mean square error (RMSE) as low as 13.875 [40]. They further delineated climatic conditions, livestock intensification levels and socioeconomic status as the predominant driving factors of brucellosis occurrence using the SHAP method in the northern Loess Plateau, the Guanzhong Plain and southern Shaanxi, respectively [40]. The long-term predictive performance of SARIMA, XGBoost and LSTM models is compared and confirms a pronounced seasonal pattern in brucellosis incidence in the Ili Prefecture, Xinjiang, which peaks in summer and autumn with the highest monthly incidence in June. The XGBoost model demonstrated superior predictive accuracy over LSTM and SARIMA, which provide a scientific basis for public health authorities to rationally allocate prevention and control resources during epidemic seasons [14]. Additionally, a hybrid KHDMDsp-LSTM model was proposed that integrates Koopman spectral analysis and LSTM networks which employ a hybrid Mercer kernel to linearize nonlinear epidemic processes and utilize elastic-net regularization to select critical dynamical modes. This framework retains the LSTM capacity for capturing nonlinear temporal dependencies. The model outperformed baseline approaches in both reconstruction and long-term prediction of monthly brucellosis data spanning 11 years across 12 cities in Inner Mongolia, revealing a stable transmission pattern centered in the central-eastern region of Inner Mongolia with gradually diminishing outward spread [40].

Compared with epidemiological models, AI integrates diverse data sources including historical incidence records, meteorological variables, livestock inventory, stocking density, vaccination coverage and regional animal movement patterns and can construct multifactorial disease risk assessment frameworks [41]. Predicting dynamic epidemic trends across different regions and time periods by learning spatiotemporal data features with automated risk stratification can achieve pre-emptive early warning of potential outbreaks. Distinct from conventional empirical approaches, AI models equipped with interpretability methods such as SHAP can quantitatively disentangle the relative contributions of environmental and anthropogenic factors to disease transmission, thereby clarifying region-specific key driving forces. Collectively, these studies demonstrate that spatiotemporal ML and enhanced LSTM architectures have been extensively applied in regional brucellosis epidemiological research. The integration of meteorological fluctuations, stocking density, livestock product trade, and immunization coverage data has improved the accuracy of medium- and short-term epidemic forecasting, providing robust data-driven support for formulating regionally tailored brucellosis control strategies and facilitating the paradigm shift from reactive response to proactive early warning in brucellosis prevention and management.

## 6. Application of AI in Pathogenesis Research and Biologics Development for Brucellosis

AI has become deeply integrated into the molecular-level dissection of pathogen–host interactions in *Brucella*. Through ML analysis of pan-genomic data, AI is being employed to resolve differences in the expression profiles of adhesins among strains originating from distinct hosts and elucidate the molecular basis of host preference [36]. AI-driven computational biology strategies have demonstrated transformative potential in the domain of biologics development. Studies have designed multi-epitope mRNA vaccine constructs targeting extracellular proteins BtuB and LptD with in silico cloning and codon optimization used to evaluate expression efficiency in prokaryotic systems [42]. AI has also facilitated the repurposing of conventional drugs with virtual screening techniques applied to identify natural compounds targeting critical *Brucella* macromolecular targets such as aminoacyl-tRNA synthetases [43]. Integrated strategies combining ML-based activity prediction with multi-scale molecular dynamics simulations have been employed to screen candidate peptides possessing both membrane-penetrating efficiency and antimicrobial activity with successful validation in intracellular infection models.

### 6.1. AI-Assisted Elucidation of Brucella Pathogenic Mechanisms and Host Interaction Patterns

The intracellular invasion and immune evasion mechanisms of *Brucella* are inherently complex [44]. *Brucella* is different from other Gram-negative bacteria in that it lacks conventional virulence factors such as exotoxins, extracellular enzymes and plasmids [45]. Instead, its pathogenicity is primarily mediated by virulence determinants including lipopolysaccharide (LPS), the type IV secretion system (T4SS) and the BvrR/BvrS two-component regulatory system, all of which collectively orchestrate host–pathogen interactions and intracellular survival [44]. Elucidating virulence gene function and host interaction mechanisms has been heavily dependent on extensive experimental validation, which results in relatively low research efficiency. Leveraging AI-based protein structure prediction and bioinformatics analysis enables rapid dissection of these key virulence systems. A model and assembled T4SS components encoded by the *vir*B operon are identified using AlphaFold in *B. melitensi*. This study identified three candidate drug molecules—Ezetimibe, Chlordiazepoxide and Alloin—exhibiting favorable ADMET properties from the DrugBank databases through virtual screening targeting the dimerization interface of the VirB11 ATPase [46]. MM-GBSA analysis and molecular dynamics simulations subsequently confirmed the stability of ligand–protein interactions, offering a drug repurposing strategy for anti-virulence therapy directed against the *Brucella* T4SS.

Concurrently, ML approaches have demonstrated advantages in mining differential gene expression data during *Brucella* infection of host cells [47,48]. Transcriptomic data integrated with ImmPort immune-related genes were analyzed through weighted gene co-expression network analysis (WGCNA), LASSO, random forest and SVM-RFE to identify key immune biomarkers [47]. These hub genes were found to be closely associated with the Th1 signaling pathway and T-cell activation, implicating their potential molecular roles in *Brucella* immune evasion and persistent infection. Wang et al. integrated WGCNA with a multi-model ML framework to identify 264 differentially expressed genes from 103 brucellosis patients and 46 healthy controls with enrichment in pathways related to mitotic cell division, chromosome segregation, cell cycle regulation and cytokine–cytokine receptor interactions. Through cross-validation using LASSO, SVM and random forest, six hub genes RTP5, KIF19, CDKN2A, RCAN2, GLB1L3 and IL12RB2 were selected, and ROC curve analysis confirmed that their combinatorial panel could serve as diagnostic biomarkers for brucellosis [47]. Immuno-infiltration analysis further revealed that brucellosis progression was positively correlated with CD8^+^T cells, follicular helper T cells and resting NK cells, which uncovers previously underappreciated immunoregulatory mechanisms. AI, particularly ML and DL, holds significant promise in multi-omics data integration and precise feature identification, which effectively bridges the gap between biomarker discovery and clinical translational applications and advances the development of precision diagnostic and therapeutic frameworks for brucellosis [7,48].

### 6.2. AI-Based Virtual Screening for Anti-Brucella Drug Discovery

The novel anti-agents represent a critical direction for addressing the challenges of antimicrobial resistance and refractory chronic infections in brucellosis. *Brucella*, as a facultative intracellular pathogen, is capable of long-term survival within macrophages. Conventional antibiotic regimens are frequently hampered by high relapse rates and the propensity for resistance acquisition, underscoring the urgent need for anti-*Brucella* drugs with novel mechanisms of action. The screening of traditional drugs relies predominantly on in vitro bacteriostatic assays, which are characterized by high costs and low throughput, rendering them insufficient to meet the pressing demands of new drug development. AI-enabled computer-aided drug design, by targeting key functional proteins of *Brucella* and performing molecular docking-based virtual screening against natural product and small-molecule compound libraries, enables the rapid identification of candidate compounds with potential anti-*Brucella* activity. This AI-driven virtual screening approach reduces the number of candidates requiring experimental validation, shortens the drug development timeline, and enhances overall R&D efficiency [49]. Elrashedy et al. employed bioinformatics methods for three-dimensional structural modeling of candidate *Brucella* proteins, including BvrR, OMP25 and OMP31 [50]. And they predicted B-cell and T-cell epitopes followed by structure-based virtual screening against the ZINC and DrugBank databases, ultimately identifying five candidate compounds exhibiting favorable ADMET profiles and no cytotoxicity, which provided a theoretical foundation for brucellosis vaccine design and drug discovery [50]. This technology could overcome the limitations of conventional empirical drug screening and provide a highly efficient technological pathway for the development of next-generation anti-brucellosis biologics, while also laying an important foundation for precision therapeutic strategies aimed at combating drug resistance and chronic *Brucella* infections.

### 6.3. AI-Assisted Vaccine Design for Brucellosis

Vaccination represents the most cost-effective and efficient measure for the prevention and control of brucellosis. However, no vaccine is currently licensed for human use against *Brucella*. Existing animal vaccines, including the S19, Rev.1, RB51, and S2 strains, are associated with residual virulence and the inherent limitation of being unable to differentiate naturally infected animals from vaccinated animals [25]. Conventional vaccine development relies heavily on empirical screening, which is characterized by protracted timelines and suboptimal efficiency. AI-driven immunoinformatics offers a novel paradigm for vaccine design, although its outputs remain computational predictions that require experimental validation.

Recent studies have employed AI-assisted multi-epitope vaccine design targeting multiple key virulence proteins and antigens of *Brucella*. In silico analyses predicted six CTL epitopes, nine HTL epitopes, seven linear B-cell epitopes and five conformational B-cell epitopes when targeting OMP31, LptE, and VirB2 [50,51]. The constructed multi-epitope vaccine was designed to be combined with the cholera toxin B subunit as a molecular adjuvant to enhance predicted immunogenicity [51]. Targeting the CcmA, CcmC, and BepC membrane proteins, an acid multi-epitope vaccine was designed with a predicted antigenicity score. Immune simulation predictions indicated a potential elevation of antibody titers, T-cell counts, and pro-inflammatory cytokine levels. In addition, AI tools were used to evaluate candidate vaccines with respect to predicted immunogenicity, allergenicity, toxicity, solubility and stability, thereby potentially reducing the high trial-and-error costs and uncontrollable safety risks inherent in traditional empirical vaccine development. Molecular docking and dynamics simulations suggested binding of the multi-epitope vaccine to TLR4 receptors, while immunoinformatic simulations (C-ImmSim) predicted proliferation of cytotoxic T lymphocytes and memory helper T cells along with significant IFN-γ production [52]. Based on these in silico validations, researchers have further used SnapGene software 7.0 to clone codon-optimized multi-epitope vaccine sequences into the pMV261 shuttle vector to construct recombinant bacillus (rBCG) plasmids, laying a foundation for subsequent vaccine production. However, it should be emphasized that these findings are derived from computational prediction and simulation; they do not constitute experimental evidence of protective immunity.

With respect to intelligent discrimination between wild-type and vaccine strains of *Brucella*, conventional serological methods are incapable of distinguishing antibodies elicited by vaccination from those induced by natural infection, severely constraining the evaluation of immunization efficacy and epidemiological traceability. Molecular detection techniques based on single nucleotide polymorphism (SNP) markers have emerged as effective tools to overcome this bottleneck. A SNP-based droplet digital PCR (ddPCR) assay was established that is capable of accurately differentiating wild-type *Brucella* strains from the S2 vaccine strain in a single reaction, achieving a detection limit of 10 copies/µL and outperforming real-time quantitative PCR [50]. The synergistic integration of AI-driven immunoinformatic epitope prediction, molecular simulation validation, and SNP-based strain discrimination technologies offers potential advantages over traditional empirical vaccine development. This integrated approach may enable efficient screening of core immunogenic regions, elimination of non-functional antigenic fragments, and optimization of vaccine architecture, with the aim of enhancing both protective efficacy and safety. Concurrently, SNP-based ML models, as representative intelligent strain discrimination tools, have overcome the long-standing challenge of serological cross-reactivity interference, providing precise support for post-vaccination immune surveillance and epidemiological source tracing. Collectively, these advances may facilitate precision immunoprophylaxis of brucellosis and accelerate the development of next-generation genetically engineered vaccines, provided that computational predictions are rigorously validated by experimental studies demonstrating protective immunity.

## 7. Current Limitations and Challenges of AI Applications in *Brucella*

Despite the preliminary achievements of AI in the diagnosis, genomic analysis, epidemic forecasting and biologics development for brucellosis, the application of AI in this field remains in its infancy with numerous bottlenecks constraining clinical translation and in-depth development. In terms of diagnosis, brucellosis is a zoonotic disease whose prevention and control require the integration of data across the human, animal and environmental spheres. However, current human clinical data remain severely siloed from veterinary surveillance and environmental data, while integration of genomic surveillance, livestock movement and earth observation data within the One Health framework is theoretically ideal. The reality of fragmented databases renders automated semantic alignment and real-time decision support practically unattainable. Moreover, testing standards across different regions and laboratories—including serological thresholds and definitions of culture positivity—remain unstandardized. Furthermore, this increases heterogeneity and bias risks in model training [53]. The scarcity of high-quality, standardized and annotated datasets poses a critical challenge. High-precision AI models depend on large volumes of standardized and multi-scenario sample data in epidemic forecasting. Currently, publicly available datasets of microscopic imaging, spectral data and genotype-phenotype matching are scarce for *Brucella* [54]. And data collection standards and quality vary considerably across laboratories, resulting in poor generalization capability and insufficient robustness for most AI models, which thus struggle to adapt to complex field application scenarios. Although a limited number of targeted studies have recently constructed small-scale datasets for intelligent *Brucella* detection and resistance prediction, industry-wide standardized datasets and public shared repositories remain absent. Thus, this constrains large-scale deployment and industrial translation of AI models. Furthermore, the poor interpretability of AI models represents a significant obstacle. Current DL and ML models can generate accurate predictive output, but they struggle to clearly articulate the internal feature extraction logic and underlying mechanisms. And virtual screening, molecular docking, molecular dynamics and ADMET prediction are approaches that can accelerate anti-*Brucella* drug discovery. However, identification of a compound with favorable docking scores or predicted ADMET properties does not demonstrate antimicrobial efficacy. The epidemiological section focuses predominantly on studies conducted in specific regions of China. AI models trained using highly localized epidemiological data may perform well locally but poorly when transferred to another population. Given the extreme complexity of *Brucella* immune evasion mechanisms, current AI models remain inadequate for translating host and pathogen interaction information into actionable clinical decision support, which limits their utility in deep biological mechanistic elucidation and clinical implementation. In addition, the translational efficiency of laboratory research findings into field applications remains suboptimal. Most existing AI studies on brucellosis remain confined to laboratory simulations and small-scale validation with a notable absence of portable intelligent detection devices and mature application systems tailored for primary-level veterinary testing facilities and livestock farms. The deep integration of AI technology with practical production and prevention efforts is insufficient, resulting in a disconnect between research outputs and real-world application. Finally, brucellosis epidemic prediction and risk assessment require the integration of multi-source data including epidemic surveillance records, livestock management logs, meteorological variables and regional animal movement patterns. However, these data are constrained by jurisdictional management boundaries and discrepancies in statistical standards, preventing full interoperability and integration, which compromises the comprehensiveness and accuracy of AI-based predictive models. Moreover, most AI-derived predictions regarding *Brucella* antimicrobial resistance, virulence gene discovery and drug screening lack corroboration through large-scale in vitro and in vivo studies, necessitating further refinement of model reliability and practical applicability. The promotion of AI-powered rapid diagnostic technologies for brucellosis and the establishment of intelligent screening systems for image-based detection, genotyping, environmental and meteorological data and epidemic big data will support the construction of integrated multimodal AI models covering diagnosis, source tracing, early warning and therapeutic guidance, thereby enabling intelligent management of brucellosis prevention and control. AI-driven basic research and biologics innovation should be deepened. Leveraging the strengths of AI in big-data and intelligent prediction, novel pathogenic mechanisms and resistance-regulatory targets in *Brucella* should be explored to accelerate the development of next-generation anti-*Brucella* drugs and multi- epitope vaccines, which provide new technological support for source-level prevention of brucellosis.

## 8. Conclusions

**Figure 4:**
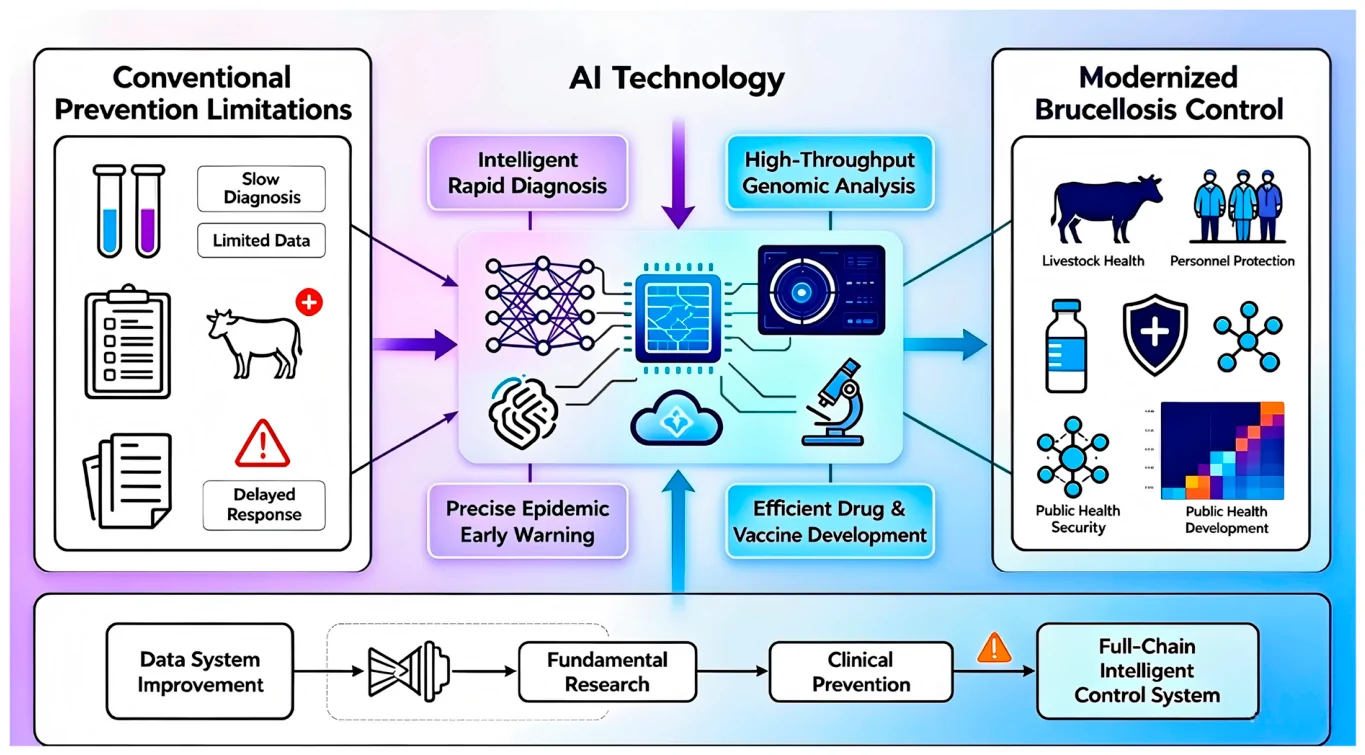
AI models may modernize brucellosis control. This figure illustrates how artificial intelligence technology can be used to overcome the limitations of conventional brucellosis prevention and control, thereby achieving modernized and comprehensive control. Faced with problems in traditional control such as slow diagnosis, limited data, and delayed response, the system introduces multiple AI technologies, including intelligent rapid diagnosis, high-throughput genomic analysis, precise epidemic early warning, and efficient drug and vaccine development. Through the application of these technologies, it is possible to significantly improve livestock health, epidemic area response capacity, and public health security, while also promoting sustainable development.

AI technologies may have the potential to address some of the limitations of conventional brucellosis prevention and control approaches. If adequately validated, these technologies could potentially offer advantages in rapid intelligent diagnosis, high-throughput genomic analysis, epidemic risk early warning, and drug and vaccine development. This could inject new momentum into the modernization and intelligent transformation of brucellosis control. At present, AI applications remain in an exploratory phase in brucellosis and are confronted with challenges including insufficient data resources, weak model interpretability and low translational efficiency. However, AI is poised to become tightly coupled with fundamental research and clinical prevention of brucellosis as data systems continue to improve. The establishment of a full-chain intelligent prevention and control system for brucellosis will provide technical support for ensuring the sustainable development of the livestock industry and public health security (Figure 4).

## Figures and Tables

**Table 1:** The concepts involved in this study.

Name	Concept	Application
**Artificial Intelligence (AI)**	Artificial intelligence refers to the overarching body of theories, methods, and technologies that enable computer systems to perform tasks that typically require human intelligence, including reasoning, learning, perception, decision-making, and natural language understanding. In biomedical research, AI is an umbrella concept encompassing machine learning, deep learning, knowledge reasoning, expert systems, and other technical approaches. It should be emphasized that AI is not equivalent to any specific algorithm—it describes the capability goal of a system rather than the means of achieving it.	1. AI-assisted pathological imaging diagnosis 2. AI-assisted intelligent interpretation of serological assays 3. AI-based epidemiological prediction and risk assessment for brucellosis 4. Application of AI in pathogenesis research and biologics development for brucellosis 5. AI-assisted vaccine design forbrucellosis
**Machine Learning (ML)**	Machine learning is a core subfield of AI, referring to methods that allow computers to automatically learn patterns from data and make predictions or decisions accordingly, without being explicitly programmed to perform a specific task. Typical ML paradigms include supervised learning (logistic regression, support vector machines, random forests), unsupervised learning (clustering, dimensionality reduction), and reinforcement learning. In clinical predictive modeling, the vast majority of so-called “AI models” actually fall within the domain of traditional ML, and their performance evaluation standards (whether external validation is required, how class imbalance is handled) differ from those of deep learning.	1. Application of machine learning in *Brucella* genomic analysis and antimicrobial resistance prediction
**Deep Learning (DL)**	Deep learning is a subset of machine learning, referring to methods that use neural networks with multiple layers (deep neural networks) to automatically learn hierarchical feature representations from raw data. Typical architectures include convolutional neural networks (CNNs), recurrent neural networks (RNNs), Transformers, and multilayer perceptrons (MLPs). The advantage of DL lies in its ability to process high-dimensional unstructured data such as images, text, and sequences in an end-to-end manner, but its sample size requirements are typically much higher than those of traditional ML, making it more prone to overfitting on small datasets.	1. Bacterial species identification (Raman scattering (SERS) analytical pipeline) 2. Detection of cells and pathogens in medical microscopic images (convolutional neural networks (CNNs) and the YOLO (You Only Look Once)) 3. Imaging diagnostic models (BSMRINet) 4. Mechanisms of infectious disease long short-term memory (LSTM) 5. Epidemiological prediction (long short-term memory (LSTM))
**Computational Approaches**	“Computational approaches” is a broader umbrella term referring to all technical means of analyzing, modeling, or inferring from biomedical data with the aid of computer algorithms, encompassing not only AI/ML/DL but also conventional bioinformatics methods including sequence alignment, phylogenetic analysis, molecular docking, statistical genetics methods and classical statistical methods including multiple regression and survival analysis.	1. Strain typing 2. Molecular traceability 3. Multi-epitope mRNA vaccine construct targeting 4. Protein structure prediction 5. Predicted B-cell and T-cell epitopes

## Data Availability

The original contributions presented in this study are included in the article. Further inquiries can be directed to the corresponding authors.
